# Human Mesenchymal Stem Cells Modulate Inflammatory Cytokines after Spinal Cord Injury in Rat

**DOI:** 10.3390/ijms150711275

**Published:** 2014-06-25

**Authors:** Lucia Machová Urdzíková, Jiří Růžička, Michael LaBagnara, Kristýna Kárová, Šárka Kubinová, Klára Jiráková, Raj Murali, Eva Syková, Meena Jhanwar-Uniyal, Pavla Jendelová

**Affiliations:** 1 Institute of Experimental Medicine, Academy of Sciences of the Czech Republic, Vídeňská 1083, Prague 14220, Czech Republic; E-Mails: urdzikl@saske.sk (L.M.U.); j.ruzicka@biomed.cas.cz (J.R.); karova@biomed.cas.cz (K.K.); sarka.k@biomed.cas.cz (S.K.); jirakova@biomed.cas.cz (K.J.); sykova@biomed.cas.cz (E.S.); 2 Department of Neuroscience, Charles University, Second Faculty of Medicine, Prague 14220, Czech Republic; 3 Department of Neurosurgery, New York Medical College, Valhalla, NY 10595, USA; E-Mails: m.labagnara@gmail.com (M.L.); Raj_Murali@NYMC.edu (R.M.); meena_jhanwar@NYMC.edu (M.J.-U.)

**Keywords:** mesenchymal stem cells, spinal cord injury, inflammatory cytokines

## Abstract

Transplantation of mesenchymal stem cells (MSC) improves functional recovery in experimental models of spinal cord injury (SCI); however, the mechanisms underlying this effect are not completely understood. We investigated the effect of intrathecal implantation of human MSC on functional recovery, astrogliosis and levels of inflammatory cytokines in rats using balloon-induced spinal cord compression lesions. Transplanted cells did not survive at the lesion site of the spinal cord; however, functional recovery was enhanced in the MSC-treated group as was confirmed by the Basso, Beattie, and Bresnahan (BBB) and the flat beam test. Morphometric analysis showed a significantly higher amount of remaining white matter in the cranial part of the lesioned spinal cords. Immunohistochemical analysis of the lesions indicated the rearrangement of the glial scar in MSC-treated animals. Real-time PCR analysis revealed an increased expression of Irf5, Mrc1, Fgf2, Gap43 and Gfap. Transplantation of MSCs into a lesioned spinal cord reduced TNFα, IL-4, IL-1β, IL-2, IL-6 and IL-12 and increased the levels of MIP-1α and RANTES when compared to saline-treated controls. Intrathecal implantation of MSCs reduces the inflammatory reaction and apoptosis, improves functional recovery and modulates glial scar formation after SCI, regardless of cell survival. Therefore, repeated applications may prolong the beneficial effects induced by MSC application.

## 1. Introduction

The spinal cord is very susceptible to injury and possesses a limited capacity to self-repair. A spinal cord injury (SCI) occurs when there is damage to the spinal cord, either as a result of trauma, the loss of its normal blood supply or compression due to a tumor or infection. Following mechanical or primary injury are several processes of secondary injury, including ischemia, hemorrhage, glutamate excitotoxicity, *etc*., that further deteriorate the initial degree of injury [1]. Treatment strategies for SCI are mostly focused on influencing such secondary damage. Mesenchymal stem cells (MSC) isolated from bone marrow display neuroprotective properties upon implantation into a spinal cord lesion [2]. This effect is due to their paracrine properties mediated by the release of growth factors, anti-apoptotic molecules and anti-inﬂammatory cytokines, creating a favorable environment for the regeneration of neurons, remyelination [3,4,5] and immunomodulation [6,7]. However, the exact mechanisms underlying their neuroprotective role is not yet fully understood. Several advantages of MSCs are evident when compared to other types of stem cells used for SCI, *i.e*., they can be easily obtained from adult donors, expanded and autologously transplanted with very low risks of malignant transformation. Furthermore, the immunomodulatory and immunosuppressive properties of MSCs have been recently evaluated in several experimental and clinical studies [8,9]. So far, the use of MSCs has been confirmed as safe in many clinical studies [10,11]; however, more information concerning their underlying mechanism is needed to optimize their application in clinical practice. The main objective of this study is to elucidate the effects of intrathecal transplantation of MSCs on the secretion of inflammatory cytokines and different neuroregenerative processes, whilst confirming the positive effects on the behavioral performance of rats after SCI.

## 2. Results and Discussion

### 2.1. Spinal Cord Injury and Cell Transplantation

**Figure 1:**
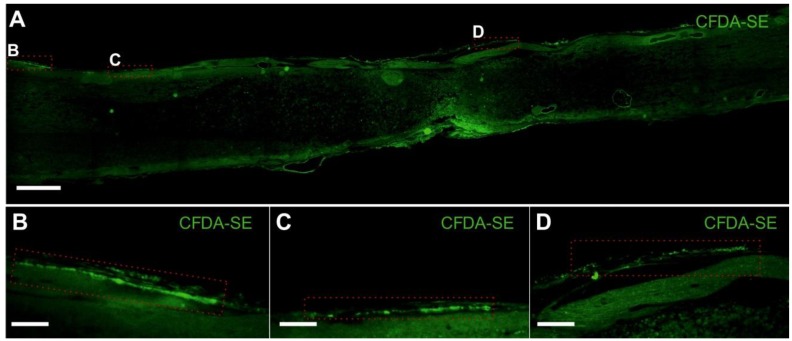
MSC survival and distribution one week after implantation. (**A**) Mosaic image of a longitudinal sectioned spinal cord with carboxy-fluorescein diacetate succinimidyl ester (CFDA-SE) labelled MSCs on the dorsal surface; (**B**–**D**) Cells attached to the spinal cord surface. Scale bars (**A**) 500 µm, (**B**–**D**) 100 µm.

Spinal cord injury was performed according to a standard protocol [2]. MSCs were transplanted intrathecally seven days after lesion induction. We did not observe any positive cells labeled with human nuclei or human mitochondria (MTC02) markers, used for the detection of human MSC in the host tissue [12,13], in the spinal cord tissue eight weeks after cell application. Consequently, we performed another set of experiments, in which the intrathecally injected cells were labeled with carboxy-fluorescein diacetate succinimidyl ester (CFDA-SE) lipophilic dye, and the rats were sacrificed one week after injection. We used CFDA-SE to avoid staining with antibodies and to reduce manipulations of slices during tissue processing. Few cells attached to the spinal cord surface (Figure 1), indicating how most of the MSCs remained in the intrathecal space, whilst the few that attached to the tissue where only able to survive for a limited period of time. Similar results were observed after the intrathecal application of magnetically-labeled rat MSC [12]. From the 0.5 million of GFP^+^ rat MSC injected intrathecally into the rats with SCI, only 720 ± 138 cells were detected in the dorsal intrathecal space seven days later. This suggests that the MSCs did not migrate into the spinal cord parenchyma. Nevertheless, their transplantation triggers several changes in the microenvironment of the spinal cord lesion, such as changes in the levels of proinflammatory cytokines, gene expression and rearrangement of the glial scar (described in the paragraphs below). These alterations, to some extent, may be transient and, therefore, may require repeated application. Our results are in agreement with the studies of Mothe *et al*., 2011, and Cizkova *et al*., 2011 [14,15], where the intrathecally-transplanted MSCs remained mostly attached to the pia mater or accumulated around the anterior spinal artery with low survival rates. Repeated intrathecal application in Cizkova’s study, which resulted in a more enhanced functional improvement and better cell survival compared to the use of single injections, was performed three times daily for three days from day seven to nine after SCI. However, based on our results, where reduced levels of inflammatory cytokines were seen up to 14 days after SCI, we believe that the cell application can be repeated once a week, for up to two weeks (for details, see below).

### 2.2. Behavioral Testing

#### 2.2.1. BBB Test

**Figure 2:**
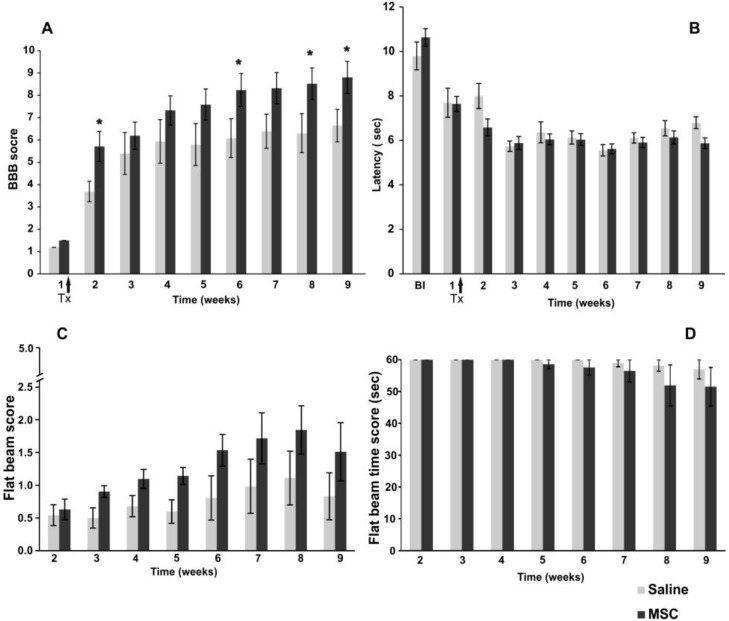
Functional recovery after spinal cord injury: (**A**) Improvement in locomotor behavior on the Basso, Beattie, and Bresnahan (BBB) scale after spinal cord injury (SCI) in control and MSC-treated groups. The MSC-transplanted rats demonstrated superior BBB scores with significant improvement, when compared to controls; (**B**) The plantar test was used to measure the effect of MSC implantation on thermal stimulation. No significant effect of implanted MSC on hyperalgesia was observed during the study; (**C**) The flat beam test evaluates the muscle strength, body and forelimb-hindlimb coordination in MSC and control groups. MSC-treated animals showed an insignificant strong trend improvement compared to the control group from the sixth week onwards after SCI; (**D**) The flat beam time score shows the ability of treated animals to move across the beam within 60 s, which was observed from the fifth week onwards in the MSC-treated group. Statistical significance is marked by *, where *p* ≤ 0.05.

Locomotor recovery following SCI was evaluated in open field with the use of the Basso, Beattie, and Bresnahan [16] test (BBB test). One week after injury (the day before MSC implantation), there was no significant difference between the two groups randomly selected before the surgery. The first week post-treatment, a significant effect in faster functional recovery was observed in animals implanted with MSCs (two-way RM (repeated measures) ANOVA, *p* < 0.05). The improvement in locomotor behavior was significant in the second week (Student–Newman–Keuls (SNK) test, *p* < 0.05), as well as from the sixth week onwards following SCI (Figure 2A) (SNK test, sixth week; *p* < 0.05, eighth week; *p* < 0.05, ninth week; *p* < 0.05). The effect of MSCs on locomotor recovery (using the BBB score) was recently described by a meta-analysis model. The animals treated with MSCs scored, on average, 3.9 points higher than untreated controls [1]. However, there was extremely high variability between the studies (improvement from 0.3 to 10 points, when compared to controls), which is connected to the different types and severities of lesion models, as well as the varying methods of MSC application [17,18,19]. In our previous studies, we applied rat MSCs directly into the lesion that had been isolated from either bone marrow [20] or adipose tissue [21]. Compared to the controls, the MSC-treated rats showed an improvement in the BBB scores, increasing by 1.9 and 2.3 points, respectively. In both studies, the cells survived in the spinal cord parenchyma till the end of the experiment. Our results from intrathecal applications are within the range of previously observed improvements, despite the fact that the grafted cells were human and did not migrate and survive in the spinal cord tissue.

#### 2.2.2. Plantar Test

Thermal nociception (hyperalgesia) after SCI was assessed by utilizing the plantar test (Ugo Basile). Animals were tested three times during the week prior to injury, and subsequently, no significant difference was observed between the randomly selected groups. Furthermore, during the initial week post-injury, no significant difference was observed between the MSC and control groups (two-way RM ANOVA, *p* >> 0.05). During the study, the latency of withdrawal for both MSC- and saline- (control) treated groups decreased in both groups during the first three weeks after injury compared to before SCI and remained significant to the end of the study (Figure 2B). The decrease in latency withdrawal is most likely due to a developed hyperalgesia connected to SCI and an adaptation of the subjects to the testing conditions (such as remembering the testing procedure). Nonetheless, our results confirm that intrathecal transplantation of MSCs does not produce any additional hypersensitivity or allodynia, both previously reported adverse effects of cell therapy [22].

#### 2.2.3. Flat Beam Test

The recovery of motor function and forelimb-hindlimb coordination was measured using the flat beam test (Figure 2C). Due to the severity of the lesion and the limited recovery of the lost locomotive function at the beginning of the testing period, most of the animals started with either no or an only partial ability to balance on the beam without any forward movement. The MSC-treated animals showed a strong, yet insignificant trend (two-way RM ANOVA, *p* = 0.13) in better performance in the flat beam test, especially from the sixth week onwards, following SCI. This correlates with the results obtained from the BBB test. The flat beam time scores (Figure 2D) reflect the time animals took to start moving across the beam within a 60-s time frame; this was observed from the fifth week onwards. These results correspond with the severity of the lesion and an average BBB score below 10, which represents the grade of body weight support. Most of the animals performed well with body balance stabilization and gain support, but were not able to walk. The majority of the animals (three out of four) that were able to cross either the whole or part of the beam were from the MSC-treated group. In control animals, only one animal was able to partly cross the beam.

### 2.3. Histology and Immunohistochemistry

#### 2.3.1. Grey/White Matter Sparing

**Figure 3:**
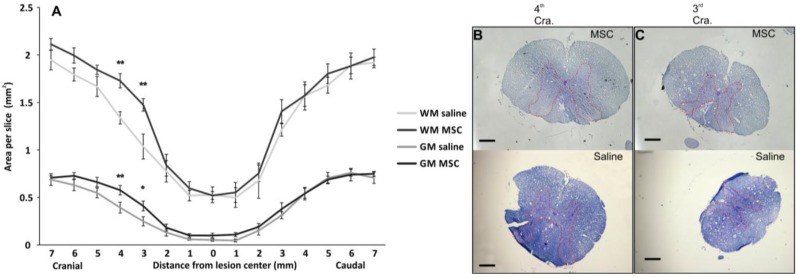
Morphometry measurement of the spinal cord lesion of spared white and gray matter in the lesion center. (**A**) MSC-transplanted rats showed significantly preserved gray matter in the whole area of interest and spatially preserved white matter; Local significance was detected particularly in the cranial direction from the central lesion part at 4-mm (**B**) and 3-mm slices (**C**). The red dotted line depicts the border of grey matter. Statisticalsignificance was marked with *, where *p* < 0.05, or with **, where *p* < 0.01.Scale bar: (**B**,**C**) 0.4 mm.

Spared white/gray matter was measured on transversal spinal sections two months after injury (Figure 3). Gray matter in the MSC-treated animals was significantly spared (two-way RM ANOVA test, *p* < 0.05), particularly 3–4 mm cranially from the lesion center (SNK test, 4 mm; *p* < 0.01, 3 mm; *p* < 0.05), which could be related to the decrease in proinflammatory cytokines (see below). The spared white matter of the MSC group significantly increased 3–4 mm cranially (SNK test, 4 mm; *p* < 0.01, 3 mm; *p* < 0.01) from the center of the lesion compared to the controls; however, a significant spatial-dependent effect in the cranial section was also observed (two-way RM ANOVA, *p* < 0.05). The prevention of tissue atrophy has been previously described by several authors as one of the actions that leads to higher behavioral recovery after MSC transplantation [17,18]. The method of MSC application plays an important role in tissue preservation, cell survival and immune response after SCI, with the intrathecal application providing superior results compared to those of intravenous or acute implantation into the lesion [23]. In the current study, the volume of white and grey matter was spared less after the intrathecal application of human MSC, compared to our previous experiment, which involved rat MSCs being injected subacutely into the lesion epicenter, as well as cranially and caudally [20]. In the latter, we reported spared white and grey matter in the whole volume of the 2 cm long spinal cord segment. However, cell application over three injections was more invasive and slowed down locomotor recovery (BBB tests showed locomotor improvement six weeks after transplantation). It must also be taken into consideration that human cells are xenograft; therefore, the results cannot be fully comparable to experiments done using rat MSCs.

#### 2.3.2. Axonal Sprouting

**Figure 4:**
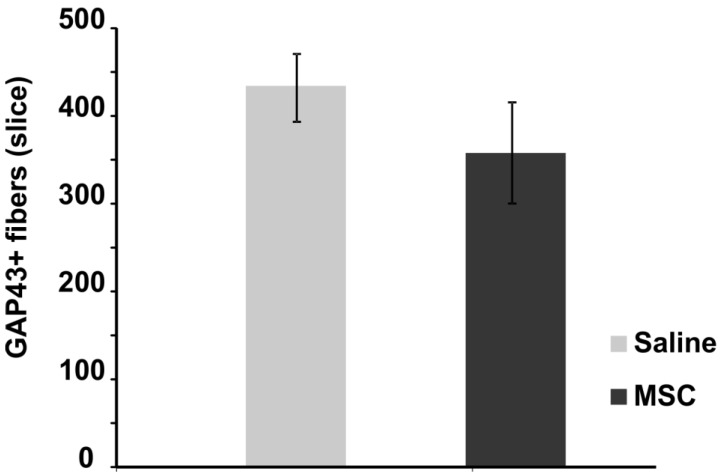
Axonal sprouting after SCI. The graph shows the effect of MSC treatment on axonal sprouting two months after injury. No significant effect of implanted MSCs was observed compared to the control group.

The number of newly sprouted axonal fibers was measured on GAP43-stained transversal spinal sections two months post-injury. Compared to the control group, no significant difference in the mean number of GAP43 positive fibers per slice was observed (Figure 4). There was an upregulation of mRNA expression of GAP43 protein (see below), which did not lead to increased axonal sprouting, this is most likely due to the short period of MSC survival and, thus, the limited growth factor support from the transplanted MSC. Robust axonal sprouting was described after intraspinal injection of spinal progenitor cells into SCI, which was most likely due to NGF growth factor produced by grafted cells [20].

#### 2.3.3. Blood Vessel and Axonal Support

Ingrowth of axons and blood vessels into the damaged spinal cord was assessed on longitudinal spinal sections two months post injury (see Supplementary Material, Figure S1). No significant difference was observed regarding the ingrowth of axons or blood vessels into the lesion. MSCs are known to support vascularization and axonal ingrowth, however, only in combination with bridging scaffolds, which serve as a permissive environment for tissue reconstruction [24,25]. Though we observed a trend towards an upregulation of mRNA expression of VEGF and CNGF growth factors in the MSC-treated group, these changes did not lead to improved revascularization of the lesion site.

#### 2.3.4. Glial Scar Re-Modulation

**Figure 5:**
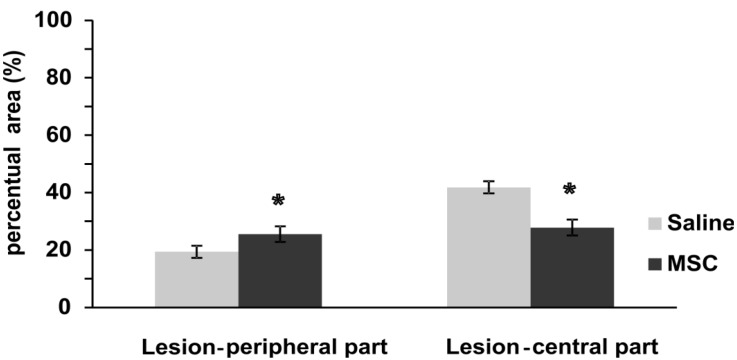
Astrogliosis after SCI. This graph depicts significant changes in the GFAP-positive area after MSC implantation. The control group showed a significantly higher signal in GFAP fluorescence in the central and lower parts in the peripheral area of the lesion center, when compared to the MSC-treated group. Statistical significance was marked with *, where *p* < 0.05.

**Figure 6:**
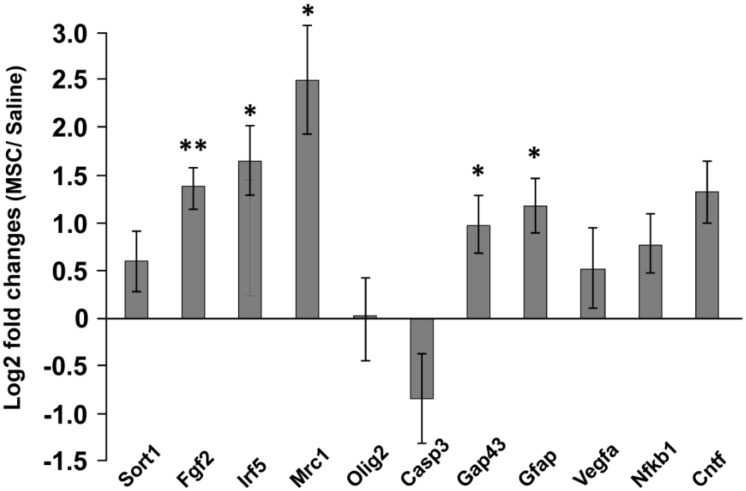
Gene expression profiling in the lesion center 28 days after SCI. The graph shows the log2 fold changes of the corresponding genes 28 days after the intrathecal transplantation of MSCs in comparison to the vehicle (saline) values. Data are represented as the mean SEM, * *p* < 0.05, ** *p* < 0.005 (Δ*C*_t_ values of MSC *vs*. vehicle).

GFAP positivity was measured on longitudinal sections two months post injury (Figure 5 and Figure S2). In MSC-treated animals, the thickness of the GFAP-positive scarring was similar in the central part of the lesion as on the periphery and was less dense (weaker GFAP staining intensity) than in the central part of the lesion in control animals. On the contrary, in control animals, the central part of the lesion was surrounded by a thick layer of glial scarring (strong GFAP positivity) (*t*-test, F = 0.69; *p* < 0.05), while the positivity was weaker at the periphery of the lesion (*t*-test, F = 0.19; *p* < 0.01). Reactive astrocytes contribute to the formation of glial scarring, through the secretion of chondroitin sulfate proteoglycans (CSPGs), and this results in the impediment of axonal regeneration. Although reactive astrocytes have long been considered detrimental to the repair of an injured spinal cord, more recent studies have shown that reactive astrocytes can support spinal cord repair [26]. Injection of embryonic stem cell-derived motor neuron progenitors or oligodendrocyte progenitors into a spinal cord transection promoted astrogliosis, through the activation of Jagged1-dependent Notch and Jak/STAT signaling, which support axonal survival [27]. In contrast, GFAP staining intensity was significantly reduced by treatment with human umbilical cord-derived MSCs [13]. In control animals with spinal cord contusion injury, GFAP immunoreactivity was detected surrounding the lesion site at four weeks post-injury, and these astrocytes were packed tightly together as a scar barrier. In contrast, astrocytic fronts were less prominent and GFAP immunoreactivity in the adjacent regions was weaker in the umbilical cord-derived MSC-grafted group. In our experiments, we have seen the upregulation of the GFAP gene four weeks after injury (Figure 6), which did not result in a dense glial scar, but rather loose scar tissue eight weeks after SCI. This glial scar remodulation suggests a greater permeability of the glial scar in MSC-treated SCI animals in comparison to the control group, which might be one of the factors playing a role in the better behavioral recovery of those treated with MSCs.

### 2.4. Gene Expression

The expressions of genes related to immune reactions (Mrc1, Irf5, Nfkb1), apoptosis (Casp3), vascularization (Vegfa), growth factors (Sort1, Fgf2, Cntf), axonal sprouting (Gap43), astrogliosis (Gfap) and oligodendrocytes (Olig2) were determined 28 days after MSC transplantation (Figure 6). 

Genes with the highest upregulation were Irf5 and Mrc1, which represent the reaction of M1 and M2 microglia/macrophages. Significant upregulation was also detected for Fgf2, the astrocytic marker, Gfap, and the marker for axonal sprouting, Gap43.

Activated microglia and recruited macrophages are among the main effector cells of the inflammatory response that follow SCI and are associated with the production of proinflammatory cytokines and related immune effector molecules that can induce both necrotic and programmed cell death, which correlate with neurological deficit [28].

Two subtypes of macrophages have become of interest in spinal cord regeneration: the proinflammatory M1 phenotype and the anti-inflammatory M2 phenotype. Recent studies have demonstrated phenotypic changes in macrophages during the immunological and inflammatory responses to various conditions. Furthermore, they have shown the beneficial and neuroprotective effect of macrophage polarization into the M2 phenotype [29,30]. SCI has been shown to be associated with a temporal M2 microglia/macrophage response, which may act as a possible repair or neuroprotective mechanism, while the increased prevalence of activated M1 microglia/macrophages may lead to neuronal loss and demyelination, despite the presence of neurotrophic factors [31].

To study the activation of the inflammatory/anti-inflammatory immune reaction, we detected the expression of Irf5 for M1 and Mrc (CD206) for M2 macrophage activation. Interferon regulatory factor 5 (Irf5) is the major regulator of proinflammatory M1 macrophage polarization, which directly induces the expression of proinflammatory cytokines, such as IL-6, IL-12b and IL-23a, whilst repressing transcription of anti-inflammatory cytokines, such as IL-10 [32]. On the other hand, M2 macrophages stimulate humoral responses, tissue remodeling and angiogenesis through the production of anti-inflammatory cytokines (IL-10).

In this study, the expression of both, Irf5 and Mrc1 increased in the spinal cord lesion 28 days after MSC transplantation, which reflects how both inflammatory, as well as anti-inflammatory responses were stimulated after treatment. Irf5 upregulation may be responsible for the rising levels of IL-6 and IL-12 cytokines in the MSC group 28 days after SCI, when compared to their levels 14 days after SCI (Figure 6).

### 2.5. Inflammatory Cytokine

**Figure 7:**
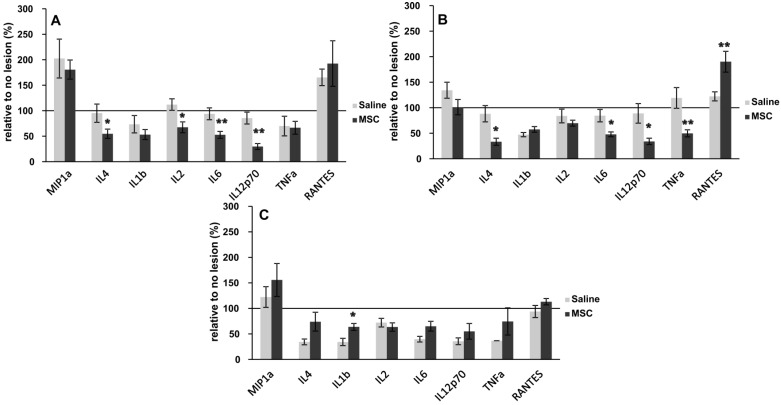
Levels of proinflammatory cytokines 10, 14 and 28 days after SCI in MSC- and control-treated rats. (**A**) Ten days after SCI, a significant decrease in the levels of IL4, IL2, IL6 and IL12p70 was observed; (**B**) Fourteen days after SCI, the decrease of IL4, IL6, IL12p70 and TNF-α continued; (**C**) Twenty eight days after SCI, the levels of cytokines did not reduce; rather, they were approximately equal to that of controls, with the exception of IL1b, where an increase in cytokine levels was seen. Statistical significance was achieved at *p < 0.05, **p < 0.01.

Levels of IL-4, IL-1β, IL-2, IL-6, IL-12, TNF-α, MIP-1α and RANTES were evaluated to determine the effects of MSC transplantation on the levels of cytokines in the SCI at 10, 14 and 28 days after lesioning. In the MSC-transplanted rats, the amounts of IL-2, IL-4, IL-6 and IL-12 reduced greatly after 10 days after SCI, and the levels of TNF-α, IL-2, IL-4, IL-6 and IL-12 were significantly lower at 14 days post-SCI, while those of RANTES were significantly higher in the MSC-transplanted rats. A different pattern was observed in the levels of cytokines at 28 day; enhanced levels of TNF-α, IL-4, IL-6 and IL-12 were observed, but not for RANTES, IL2 and MIP1a, which demonstrated lower or unchanged levels (Figure 7).

Our results, in part, are in agreement with the findings of Nakajima *et al*., 2012 [33], as they also show a decrease in cytokine levels one week after MSC transplantation. Cytokines are an important regulator of inflammation following acute SCI. Evidence suggests that MSC transplantation results in increased levels of IL-4 and IL-14, with concomitant decreases in TNF-α and IL-6 within the first week [33]. Our results somewhat support these findings and build upon them. A significant decrease in IL-4, IL-2, IL-6 and IL-12p70 10 days after treatment was reported (Figure 6A). Levels of IL-4, IL-6 and IL-12p70 remained suppressed relative to controls even after 14 days of MSC treatment. However, levels of TNF-α were markedly decreased compared to controls, and there was a significant increase in RANTES. Locomotor improvement as assessed by the BBB scores obtained 14 days after SCI began to trend toward MSC treatments. Levels of IL-4, IL-6 and TNF-α had all increased in MSC-treated rats by 28 days. BBB scores were again higher in treated animals, suggesting that these cytokines are increasingly important in the later stages of healing. These findings suggest that TNF-α is deleterious in the early phase of healing, while RANTES may have a beneficial effect. 

Whilst the exact mechanism by which MSCs modulate cytokine levels remains to be fully understood, based on available evidence, it appears that grafted MSCs can modify the inflammatory environment by shifting the macrophage phenotype from M1 to M2 and by reducing the levels of TNF-*α* and other inflammatory cytokines. Therefore, MSCs may modulate the immune response and induce anti-inflammatory effects [34,35,36]. Furthermore, the availability of MSCs for autologous and allogeneic transplantation makes them useful in the treatment of SCI. 

## 3. Experimental

### 3.1. Animals

To complete this study, 66 ten-week-old male Wistar rats were used. Their body weight was controlled at 300 ± 15 g to minimize differences in body size, in order to achieve standardized spinal cord lesions. The rats were housed in pairs and maintained at 22 °C and on a 12:12 h light: dark cycle. Food and water were provided *ad libitum*. Seven days after injury, all animals were implanted intrathecally either with MSCs (*n* = 38) or saline (*n* = 28). Before SCI, animals were separated into two groups; the first set of animals contained those rats analyzed using Luminex (cytokine detection) and qPCR evaluation. For each time point (10, 14 and 28 days after injury), five rats were used per group. The second set of animals contained those that survived either two or eight weeks after lesioning and were injected with CDFA-SE-labeled cells or evaluated using behavioral studies, as well as histological and immunohistochemical methods (MSC *n* = 23, saline *n* = 13). Histological and immunohistochemical evaluation was performed either on spinal longitudinal (MSC *n* = 16, saline *n* = 7) or cross (MSC *n* = 7, saline *n* = 6) -sections.

All experiments were performed in accordance with the European Communities Council Directive of 24 November 1986 (86/609/EEC), regarding the use of animals in research and were approved by the Ethics Committee of the Institute of Experimental Medicine Academy of Sciences Czech Republic, Prague, Czech Republic

#### 3.1.1. Spinal Cord Injury and Cell Transplantation

Balloon-induced spinal cord compression lesions were used as the model of spinal cord injury in rats [2]. After the induction of isoflurane anesthesia, a 2-French Fogarty catheter was inserted into the epidural space through laminectomy at vertebrae T10, in rats weighing 300–330 g. Body temperature was maintained at 37 °C during the whole surgical procedure. Spinal cord compression was induced by the inflation of the balloon with 15 microliters of saline for 5 min at the T8 spinal level. After lesion induction, the catheter was removed, and the wound was sutured in anatomical layers. Gentamicin (Lek Pharmaceutical 5 mg/kg) was given intramuscularly to prevent post-surgery infection. Manual bladder expression was performed twice per day. After spinal cord injury, rats were randomly selected into two groups for saline (*n* = 28) or MSC (*n* = 38) treatment. Seven days after spinal cord injury, naive MSCs (*n* = 33) or those labeled with CFDA-SE (*n* = 5) were injected into the subdural space through the L5-L6 intervertebral space according to De la Calle [37]. Immunosuppression cyclosporine A (10 mg/kg) and azathioprine sodium (2 mg/kg) were used to prevent the rejection of the cell transplants and were used in both groups of animals.

Human mesenchymal stem cells were obtained from the company, Bioinova. All MSCs preparation procedures were performed under GMP conditions. The mononuclear fraction containing MSCs was separated from the bone marrow by gradient centrifugation using 25% Gelofusine (B. Braun, Melsungen, Germany). The cells were expanded in media containing Alpha MEM Eagle without deoxyribonucleotides, ribonucleotides and UltraGlutamin (Lonza, Basel, Switzerland) supplemented with 5% mixed allogeneic thrombocyte lysate (Bioinova, Prague, Czech Republic) and 10 μg/mL gentamicin (Lek Pharmaceuticals, Ljublanja, Slovenia); non-adherent cells were washed out by changing the medium. Cells from the second passage were analyzed and used for transplantation. The expression of specific surface markers was assessed using fluorescent-activated cell sorting (FACS) analysis (FACSAria flow cytometer, BD Biosciences, San Diego, CA, USA). The cells were positive for the following markers, CD105, CD73 and CD90, and negative for CD45, CD34, CD14 or CD11b, CD79alpha and HLA-DR (Human Leukocyte Antigens locus DR) surface molecules [38].

To detect the distribution and survival of implanted cells, five rats with SCI were implanted with MSCs freshly labeled with fluorescent dye (10 µg carboxy-fluorescein diacetate succinimidyl ester (CFDA-SE, Green Fluorescence, Molecular Probes, Eugene, OR, USA)). The labeling was performed according to the manufacturer’s protocol.

### 3.2. Behavioral Testing

#### 3.2.1. BBB Test

Locomotor ability was evaluated using the Basso, Beattie and Bresnahan test [16]. Two independent examiners studied the locomotor ability of experimental rats for approximately four consecutive minutes once a week, starting in the first week after injury. The animals were placed on a floor within a circular enclosure. Their hindlimb joint movement, paw placement, weight support, forelimb-hindlimb coordination, *etc*., were evaluated according to a scale from 0–21. 

#### 3.2.2. Plantar Test

The plantar test was performed using a standard Ugo Basile test apparatus (Ugo Basile, Comerio, Italy) on MSC- or saline-treated rats. The test consisted of placing a rat in a transparent acrylic box; a mobile infrared heat lamp was positioned underneath the targeted hind paw. A thermal radiant stimulus was then applied to the plantar surface, and the latency of the paw withdrawal response was measured automatically with the help of a photoelectric-sensitive device. The latency of the withdrawal response of each hind paw was determined before and after the SCI was induced. The test was performed weekly after SCI, during a given time period. Each paw was stimulated five times. Hyperalgesia in response to heat was defined as a significant decrease in withdrawal latency.

#### 3.2.3. Flat Beam Test

Motor function and forelimb-hindlimb coordination was assessed using the flat beam test on MSC- or saline-treated rats. The apparatus consists of a 3.4 cm-wide by 140 cm-long wooden rectangular beam. A goal box was placed at one end. The central part of the beam (1 m long) was used to evaluate the walking distance. The latency and the trajectory of the rat to traverse the beam were recorded with a video-tracking system (TSE-Systems Inc., Bad Homburg, Germany) for a maximum of 60 s. After pre-training, two trials were given each day for three consecutive days. The animals were examined before surgery, then every week from the second week after lesion induction. A 0- to 5-point scale modified from Goldstein [39] was used to evaluate the locomotor function, starting with no ability to balance and progressing to crossing the whole length of the beam properly using both hindlimbs.

### 3.3. Histology and Immunohistochemistry

Two weeks after injury, the animals (*n* = 5) with the applied CFDA-SE-labeled MSCs were sacrificed (transcardial perfusion with 4% paraformaldehyde in PBS) and their spinal cords were removed. A 2 cm-long segment of the spinal cord was dissected between 1 cm cranial and 1 cm caudal to the injury epicenter. Spinal cords were longitudinally cut and mounted with no additional processing. Two months after injury, the animals with unlabeled MSCs (*n* = 18) were sacrificed (transcardial perfusion with 4% paraformaldehyde in PBS), and their spinal cords were removed. A 2 cm-long segment of the spinal cord was dissected between 1 cm cranial and 1 cm caudal to the injury epicenter. Spine samples were separated into two groups; serial longitudinal (20 μm thickness) or cross-sections (5 μm thickness). Slices were stained for preferable markers, mounted and overlaid with cover glass. To distinguish the white and gray matter Cresyl violet (0.25 g of cresyl violet dissolved in 100 mL of distilled water with 1 mL of 10% acetic acid) and Luxol-fast blue (1 g of Luxol-fast blue dissolved in 100 mL of 96% ethanol with 5 mL of 10% acetic acid) were used. Seven sections were selected at 1-mm intervals along the cranio-caudal axis from the lesion center, and whole images of the spinal cord were taken with an Axioskop 2 plus microscope (Zeiss, Oberkochen, Germany). Grey and white matter were depicted and analyzed using ImageJ software (Wayne Rasband, National Institutes of Health, Bethesda, MD, USA). For immunohistochemical analysis, the primary antibodies against GAP43, NF160, RECA and GFAP-CY3 (Protocol, Sigma, St. Louis, MO, USA) were used. To visualize primary antibody reactivity, appropriate secondary antibodies were used: goat anti-mouse IgG conjugated with Alexa-Fluor 488 (GAP43), as well as 594 (NF160, RECA) (Molecular Probes, Eugene, OR, USA). The histology and immunochemistry evaluation was carried out using a ZEISS AXIO Observer D1 microscope (Carl Zeiss, Weimar, Germany). Either Wizzard (Carl Zeiss, Germany) or ImageJ programs were used for image analysis of the histological and immunohistochemical staining. For graphics, Excel (Office 2010, Microsoft) and CorelX6 (Corel Corporation) were used. 

#### 3.3.1. Grey/White Matter Sparing

White/gray matter tissue sparing after SCI was evaluated in transversal spinal sections within the center of the lesion (±7 mm) of MSC- (*n* = 7) or saline- (*n* = 6) treated rats. The amount of spared tissue (mm^2^) and the grey/white matter ratio was measured with ImageJ software, and the arithmetical mean of each spinal section of the MSC-treated group was compared to the control group.

#### 3.3.2. Axonal Sprouting

The number of newly sprouted axonal fibers after SCI, in both animal groups, was evaluated on anti-GAP43-stained transversal sections of MSC- (*n* = 7) or saline- (*n* = 6) treated rats. High magnification images of transversal sections, separated by a 1-mm distance and stained for GAP43, were taken from both animal groups, and GAP43-positive fibers were manually counted. Each counted fiber was marked by the color red to avoid double counting.

#### 3.3.3. Axonal Ingrowth

The influence of MSC transplantation on axonal growth and survival after SCI was evaluated on longitudinal sections using NF160 staining of MSC (*n* = 11) or saline (*n* = 7) treated rats. The NF160 fluorescent signal area was calculated using Wizzard. Five images from each of the three sections were evaluated (2× cranial lesion border, 1× center of lesion, 2× caudal lesion border). Images are enclosed in the Supplementary Material (Figure S1A). The arithmetical means of NF160-positive square areas, the central part normalized per 0.38 mm^2^, were compared between groups.

#### 3.3.4. Blood Vessel Ingrowth

The influence of MSC transplantation on blood vessel lesion infiltration was evaluated on longitudinal sections using RECA (Reacts with Endothelial Cell Antigen) staining of MSC- (*n* = 11) or saline- (*n* = 7) treated rats. The RECA fluorescent signal area was calculated using Wizzard. Five images from each of the three sections were evaluated (2× cranial lesion border, 1× center of lesion, 2× caudal lesion border). Images are enclosed in the Supplementary Material (Figure S1B). The arithmetical means of RECA-positive square areas, the central part normalized per 0.38 mm^2^, were compared between groups.

#### 3.3.5. Glial Scar

The influence of MSC transplantation on glial scar after SCI was evaluated on longitudinal sections using GFAP-CY3 staining of MSC- (*n* = 11) or saline- (*n* = 7) treated rats. The GFAP-CY3 fluorescent signal area was calculated using Wizzard. Five images from each three sections were evaluated (2× cranial lesion border, 1× center of lesion, 2× caudal lesion border). The arithmetical means of GFAP-CY3-positive square areas were compared between groups and the central part was normalized per 0.38 mm^2^ (Figure S2). 

### 3.4. Gene Expression

The expression of rat target genes (Nt3 (Sort1), Fgf2, Irf5, Mrc1, Olig2, Cas3, Gap43, Gfap and Vegfa) were studied using quantitative real-time reverse transcription polymerase chain reaction (qPCR), 28 days after MSC transplantation (MSC *n* = 5, saline *n* = 5). RNA was isolated from paraffin-fixed tissue sections using the High Pure RNA Paraffin Kit (Roche, Penzberg, Germany), following the manufacturer’s recommendations. RNA amounts were quantified using a spectrophotometer (NanoPhotometerTM P-Class, Munchen, Germany). The isolated RNA was reverse transcribed into cDNA using Transcriptor Universal cDNA Master (Roche) and a thermal cycler (T100™ Thermal Cycler, Bio-Rad, Hercules, CA, USA). The qPCR reactions were performed using cDNA solution, FastStart Universal Probe Master (Roche, Germany) and TagMan^®^ Gene Expression Assays (Life Technologies, Carlsbad, CA, USA): Gapdh/Rn01775763_g1, Sort1/Rn01521847_m1, Fgf2/Rn00570609_m1, Irf5/Rn01500522_m1, Mrc1/Rn01487342_m1, Olig2/Rn01767116_m1, Cas3/Rn00563902_m1, Gap43/Rn01474579_m1. The qPCR was carried out in a final volume of 10 μL containing 25 ng of extracted RNA. Amplification was performed on the StepOnePlus™ real-time PCR cycler (Life Technologies). All amplifications were run under the same cycling conditions: 2 min at 50 °C, 10 min at 95 °C, followed by 40 cycles of 15 s at 95 °C and 1 min at 60 °C. All samples were run in duplicate, and a negative control was included in each array. Relative quantification of gene expression was determined using the ΔΔ*C*_t_ method. Results were analyzed with StepOnePlus^®^ software. The gene expression level was normalized based on Gapdh as a reference gene; control samples with vehicle (PBS) transplantation were used as a calibrator. 

The statistical significance of differences in Δ*C*_t_ values between the transplanted and control (vehicle) groups were determined using *t*-tests. Differences were considered statistically significant if *p* < 0.05. Data are expressed as the means ± the standard error of mean.

### 3.5. Cytokines

The levels of inflammatory cytokines were determined to examine the effects of MSC transplantation on the levels of cytokines at the region of the lesion, 10, 14 and 28 days after SCI (MSC *n* = 5 per time point, saline *n* = 5 per time point). A portion of the spinal cord at the site of the lesion was dissected and incubated in cell media DMEM (Sigma) supplemented with 10% FBS and 0.2% primocin. To estimate the levels of secretary cytokine levels, the media was collected at 24 h after incubation. Inflammatory cytokines were analyzed using a Customized Milliplex inflammatory cytokine kit (Millipore, Billerica, MA, USA) and Magpix instrumentation software. Rat cytokine Luminex custom 8-plex kits (for IL-2, IL-4, IL-6, IL-8, IL-10, IL-12p70, IFNγ, TNFα, MIP-1α) were used for customized bead assays. The assays were done in 96-well filter bottom plates according to the protocol. Antibody conjugated beads were used at a concentration of 5000 beads per marker, following the manufactures’ protocol. Biotinylated detection antibody was used with streptavidin-RPE (streptavidin-R-Phycoerythrin) to measure the levels of cytokines on the Luminex xMAP 200 systems and analyzed using Magpix instrumentation software. The raw data, consisting of mean fluorescence intensity (MFI), was used to calculate the concentration of each cytokine; a four- or five-parameter logistic fit curve was generated for each cytokine from the seven standards. 

The lower limit of quantification (LLOQ) was determined using the lowest standard that was at least three-times above background. The calculation of the LLOQ was performed by subtracting the MFI of the background (diluent) from the MFI of the lowest standard concentration and back-calculating the concentration from the standard curve.

### 3.6. Statistical Evaluation

The statistical significance of differences between the MSC- and saline-injected groups were determined using *t*-tests. In the case of repeated measurement tests (behavioral tests) or the spatial distribution of the treatment effect (grey/white matter sparing), two-way repeated measurement (RM) ANOVA with Student–Newman–Keuls (SNK test) *post hoc* pair-to-pair test was used (Sigmastat 3.1, Sistat Software Inc., San Jose, CA, USA). Differences were considered statistically significant if *p* < 0.05. Data are expressed as the means ± the standard error of mean.

## 4. Conclusions

A single intrathecal implantation of human MSCs seven days after SCI improves functional recovery via the reduction of the inflammatory reaction and apoptosis, as well as the modulation of glial scar formation. All of these changes are induced by cell application, even though the cells do not survive in the lesion. The decreased levels of inflammatory cytokines may be prolonged by repeated applications of the cells.

## Supplementary Files

Supplementary File 1

## References

[1] Oliveri R.S., Bello S., Biering-Sorensen F. (2014). Mesenchymal stem cells improve locomotor recovery in traumatic spinal cord injury: Systematic review with meta-analyses of rat models. Neurobiol. Dis..

[2] Urdzikova L., Jendelova P., Glogarova K., Burian M., Hajek M., Sykova E. (2006). Transplantation of bone marrow stem cells as well as mobilization by granulocyte-colony stimulating factor promotes recovery after spinal cord injury in rats. J. Neurotrauma.

[3] Coutts M., Keirstead H.S. (2008). Stem cells for the treatment of spinal cord injury. Exp. Neurol..

[4] Garbuzova-Davis S., Willing A.E., Saporta S., Bickford P.C., Gemma C., Chen N., Sanberg C.D., Klasko S.K., Borlongan C.V., Sanberg P.R. (2006). Novel cell therapy approaches for brain repair. Prog. Brain Res..

[5] Joyce N., Annett G., Wirthlin L., Olson S., Bauer G., Nolta J.A. (2010). Mesenchymal stem cells for the treatment of neurodegenerative disease. Regen. Med..

[6] Hardy S.A., Maltman D.J., Przyborski S.A. (2008). Mesenchymal stem cells as mediators of neural differentiation. Curr. Stem Cell Res. Ther..

[7] Nishio Y., Koda M., Kamada T., Someya Y., Yoshinaga K., Okada S., Harada H., Okawa A., Moriya H., Yamazaki M. (2006). The use of hemopoietic stem cells derived from human umbilical cord blood to promote restoration of spinal cord tissue and recovery of hindlimb function in adult rats. J. Neurosurg. Spine.

[8] English K. (2013). Mechanisms of mesenchymal stromal cell immunomodulation. Immunol. Cell Biol..

[9] Stagg J., Galipeau J. (2013). Mechanisms of immune modulation by mesenchymal stromal cells and clinical translation. Curr. Mol. Med..

[10] Weiss D.J., Casaburi R., Flannery R., LeRoux-Williams M., Tashkin D.P. (2013). A placebo-controlled, randomized trial of mesenchymal stem cells in copd. Chest.

[11] Karussis D., Karageorgiou C., Vaknin-Dembinsky A., Gowda-Kurkalli B., Gomori J. M., Kassis I., Bulte J.W., Petrou P., Ben-Hur T., Abramsky O. (2010). Safety and immunological effects of mesenchymal stem cell transplantation in patients with multiple sclerosis and amyotrophic lateral sclerosis. Arch. Neurol..

[12] Vanecek V., Zablotskii V., Forostyak S., Ruzicka J., Herynek V., Babic M., Jendelova P., Kubinova S., Dejneka A., Sykova E. (2012). Highly efficient magnetic targeting of mesenchymal stem cells in spinal cord injury. Int. J. Nanomed..

[13] Zhilai Z., Hui Z., Anmin J., Shaoxiong M., Bo Y., Yinhai C. (2012). A combination of taxol infusion and human umbilical cord mesenchymal stem cells transplantation for the treatment of rat spinal cord injury. Brain Res..

[14] Cizkova D., Novotna I., Slovinska L., Vanicky I., Jergova S., Rosocha J., Radonak J. (2011). Repetitive intrathecal catheter delivery of bone marrow mesenchymal stromal cells improves functional recovery in a rat model of contusive spinal cord injury. J. Neurotrauma.

[15] Mothe A.J., Bozkurt G., Catapano J., Zabojova J., Wang X., Keating A., Tator C.H. (2011). Intrathecal transplantation of stem cells by lumbar puncture for thoracic spinal cord injury in the rat. Spinal Cord.

[16] Basso D.M., Beattie M.S., Bresnahan J.C. (1995). A sensitive and reliable locomotor rating scale for open field testing in rats. J. Neurotrauma.

[17] Hodgetts S.I., Simmons P.J., Plant G.W. (2013). Human mesenchymal precursor cells (stro-1(+)) from spinal cord injury patients improve functional recovery and tissue sparing in an acute spinal cord injury rat model. Cell Transplant..

[18] Ritfeld G.J., Nandoe Tewarie R.D., Vajn K., Rahiem S.T., Hurtado A., Wendell D.F., Roos R.A., Oudega M. (2012). Bone marrow stromal cell-mediated tissue sparing enhances functional repair after spinal cord contusion in adult rats. Cell Transplant..

[19] Urdzikova L., Vanicky I. (2006). Post-traumatic moderate systemic hyperthermia worsens behavioural outcome after spinal cord injury in the rat. Spinal Cord.

[20] Amemori T., Jendelova P., Ruzickova K., Arboleda D., Sykova E. (2010). Co-transplantation of olfactory ensheathing glia and mesenchymal stromal cells does not have synergistic effects after spinal cord injury in the rat. Cytotherapy.

[21] Arboleda D., Forostyak S., Jendelova P., Marekova D., Amemori T., Pivonkova H., Masinova K., Sykova E. (2011). Transplantation of predifferentiated adipose-derived stromal cells for the treatment of spinal cord injury. Cell. Mol. Neurobiol..

[22] Pourheydar B., Joghataei M.T., Bakhtiari M., Mehdizadeh M., Yekta Z., Najafzadeh N. (2012). Co-transplantation of bone marrow stromal cells with schwann cells evokes mechanical allodynia in the contusion model of spinal cord injury in rats. Cell J..

[23] Paul C., Samdani A.F., Betz R.R., Fischer I., Neuhuber B. (2009). Grafting of human bone marrow stromal cells into spinal cord injury: A comparison of delivery methods. Spine.

[24] Hejcl A., Ruzicka J., Kapcalova M., Turnovcova K., Krumbholcova E., Pradny M., Michalek J., Cihlar J., Jendelova P., Sykova E. (2013). Adjusting the chemical and physical properties of hydrogels leads to improved stem cell survival and tissue ingrowth in spinal cord injury reconstruction: A comparative study of four methacrylate hydrogels. Stem Cells Dev..

[25] Hejcl A., Sedy J., Kapcalova M., Toro D.A., Amemori T., Lesny P., Likavcanova-Masinova K., Krumbholcova E., Pradny M., Michalek J. (2010). Hpma-rgd hydrogels seeded with mesenchymal stem cells improve functional outcome in chronic spinal cord injury. Stem Cells Dev..

[26] Renault-Mihara F., Okada S., Shibata S., Nakamura M., Toyama Y., Okano H. (2008). Spinal cord injury: Emerging beneficial role of reactive astrocytes' migration. Int. J. Biochem. Cell Biol..

[27] Lukovic D., Moreno Manzano V., Stojkovic M., Bhattacharya S.S., Erceg S. (2012). Concise review: Human pluripotent stem cells in the treatment of spinal cord injury. Stem Cells.

[28] Hausmann O.N. (2003). Post-traumatic inflammation following spinal cord injury. Spinal Cord.

[29] Cusimano M., Biziato D., Brambilla E., Donega M., Alfaro-Cervello C., Snider S., Salani G., Pucci F., Comi G., Garcia-Verdugo J.M. (2012). Transplanted neural stem/precursor cells instruct phagocytes and reduce secondary tissue damage in the injured spinal cord. Brain.

[30] Ren Y., Young W. (2013). Managing inflammation after spinal cord injury through manipulation of macrophage function. Neural Plast..

[31] Hirai T., Uchida K., Nakajima H., Guerrero A.R., Takeura N., Watanabe S., Sugita D., Yoshida A., Johnson W.E., Baba H. (2013). The prevalence and phenotype of activated microglia/macrophages within the spinal cord of the hyperostotic mouse (twy/twy) changes in response to chronic progressive spinal cord compression: Implications for human cervical compressive myelopathy. PLoS One.

[32] Krausgruber T., Blazek K., Smallie T., Alzabin S., Lockstone H., Sahgal N., Hussell T., Feldmann M., Udalova I.A. (2011). Irf5 promotes inflammatory macrophage polarization and th1-th17 responses. Nat. Immunol..

[33] Nakajima H., Uchida K., Rodriguez Guerrero A., Watanabe S., Sugita D., Takeura N., Yoshida A., Long G., Wright K., Johnson E. (2012). Transplantation of mesenchymal stem cells promotes the alternative pathway of macrophage activation and functional recovery after spinal cord injury. J. Neurotrauma.

[34] Greish S., Abogresha N., Abdel-Hady Z., Zakaria E., Ghaly M., Hefny M. (2012). Human umbilical cord mesenchymal stem cells as treatment of adjuvant rheumatoid arthritis in a rat model. World J. Stem Cells.

[35] Kim J., Hematti P. (2009). Mesenchymal stem cell-educated macrophages: A novel type of alternatively activated macrophages. Exp. Hematol..

[36] Li M., Ikehara S. (2013). Bone-marrow-derived mesenchymal stem cells for organ repair. Stem Cells Int..

[37] De la Calle J.L., Paino C.L. (2002). A procedure for direct lumbar puncture in rats. Brain Res. Bull..

[38] Machova Urdzikova L., Sedlacek R., Suchy T., Amemori T., Ruzicka J., Lesny P., Havlas V., Sykova E., Jendelova P. (2014). Human multipotent mesenchymal stem cells improve healing after collagenase tendon injury in the rat. Biomed. Eng. Online.

[39] Goldstein B., Little J.W., Harris R.M. (1997). Axonal sprouting following incomplete spinal cord injury: An experimental model. J. Spinal Cord Med..

