# Supplementary material for: Human Mesenchymal Stem Cells Modulate Inflammatory Cytokines after Spinal Cord Injury in Rat
Source: Int J Mol Sci. 2014 Jun 25;15(7):11275–93. doi: 10.3390/ijms150711275 (PMC4139782; doi:10.3390/ijms150711275)

Supplementary Information

Figure S1. Histological image of axonal and blood vessel ingrowth after SCI. The NF160 (**A**) and RECA (**B**) fluorescent signal area was calculated using Wizzard. Five images from each three sections were evaluated (2× cranial lesion border, 1× center of lesion, 2× caudal lesion border). The arithmetical means of NF160 or RECA positive square areas were compared between groups and the central part was normalized per 0.38 mm^2. Scale bar (**A**,**B**) 0.5 mm.

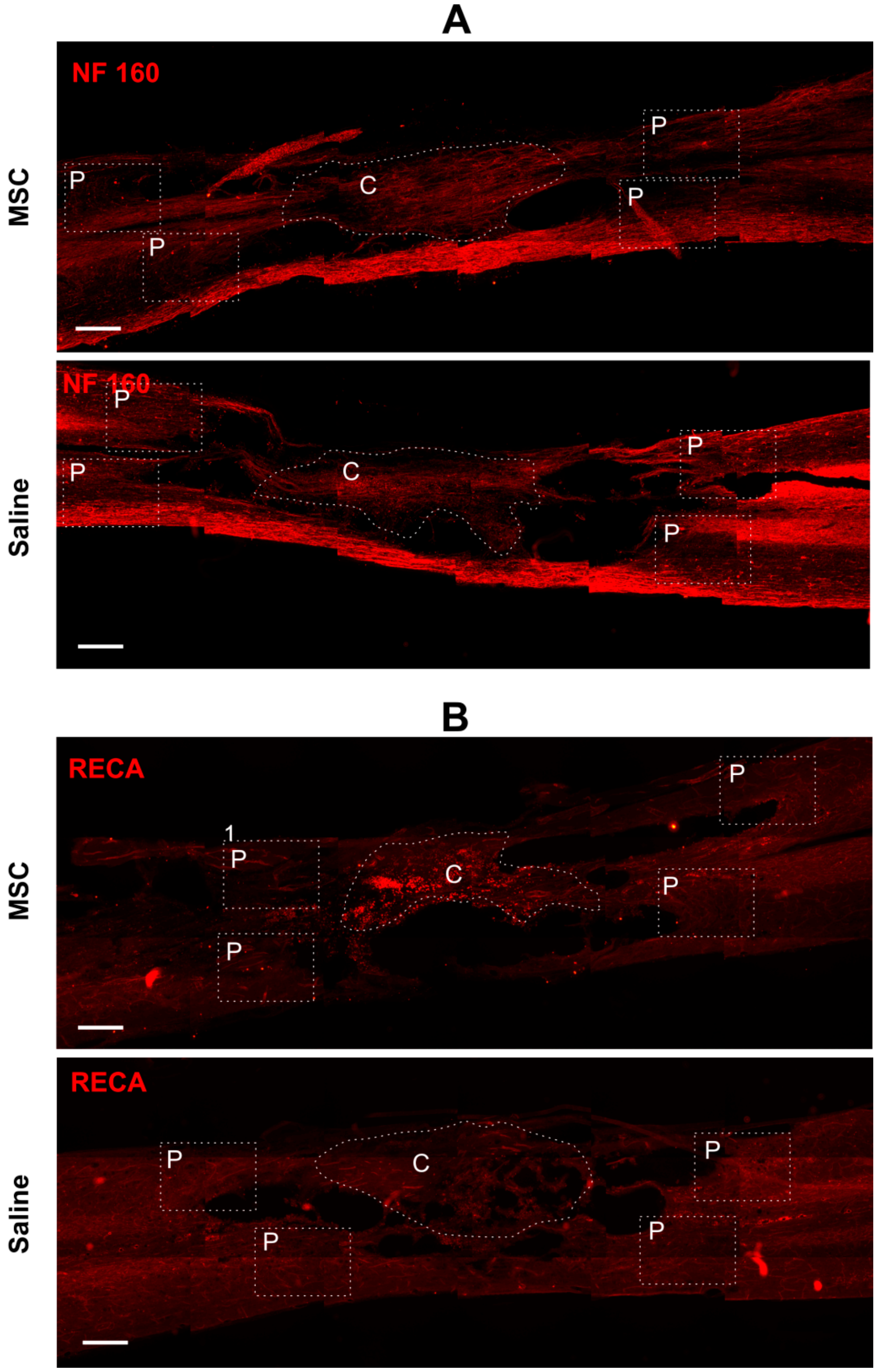

Figure S2. Histological image of astrogliosis measurements after SCI. The GFAP-CY3 fluorescent signal area was calculated using Wizzard from longitudinal sections stained for GFAP. Five images from each three sections were evaluated (2× cranial lesion border, 1× center of lesion, 2× caudal lesion border). The arithmetical means of GFAP-CY3 positive square areas were compared between groups and the central part was normalized per 0.38 mm^2. Scale bar 0.5 mm.

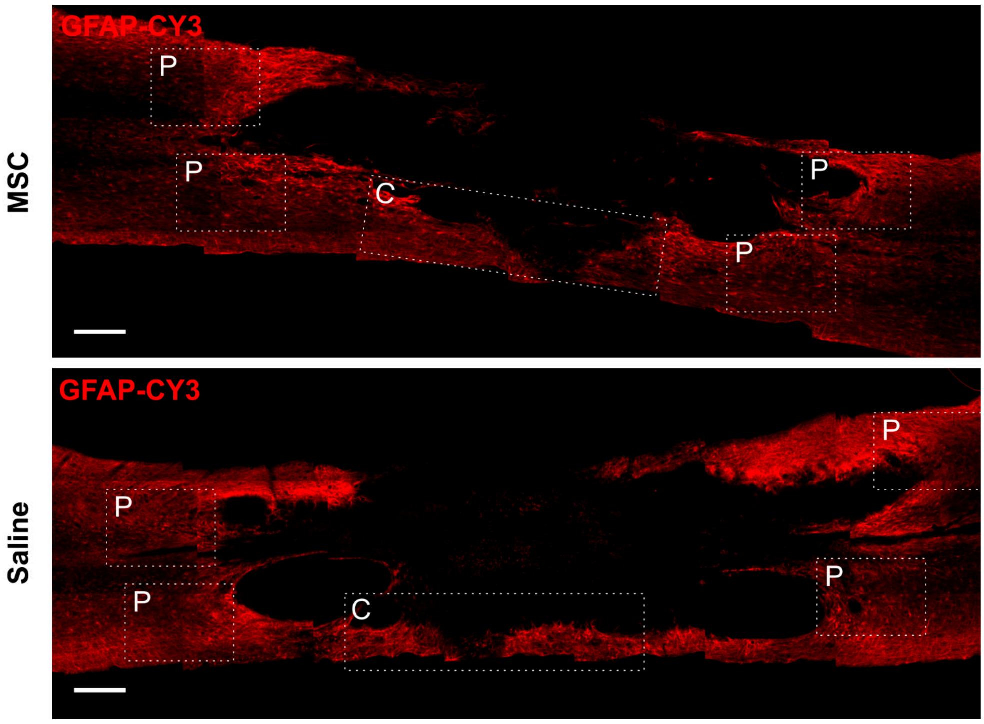

Supplement: Supplementary File 1 [file ijms-15-11275-s001.pdf]
